# Transcriptional Analysis of *Acinetobacter* sp. *neg1* Capable of Degrading Ochratoxin A

**DOI:** 10.3389/fmicb.2016.02162

**Published:** 2017-01-09

**Authors:** Vania C. Liuzzi, Francesca Fanelli, Mariana Tristezza, Miriam Haidukowski, Ernesto Picardi, Caterina Manzari, Claudia Lionetti, Francesco Grieco, Antonio F. Logrieco, Michael R. Thon, Graziano Pesole, Giuseppina Mulè

**Affiliations:** ^1^Institute of Sciences of Food Production, National Research CouncilBari, Italy; ^2^Institute of Sciences of Food Production, National Research CouncilLecce, Italy; ^3^Department of Biosciences, Biotechnologies and Biopharmaceutics, University of BariBari, Italy; ^4^Institute of Biomembranes and Bioenergetics, National Research CouncilBari, Italy; ^5^Center Hispano-Luso de Investigaciones Agrarias, University of SalamancaSalamanca, Spain; ^6^Consorzio Interuniversitario BiotecnologieTrieste, Italy

**Keywords:** peptidase, phenylalanine, mycotoxin, pathway analysis, food safety

## Abstract

Ochratoxin A (OTA) is a nephrotoxic and potentially carcinogenic mycotoxin produced by several species of *Aspergillus* and *Penicillium*, contaminating grapes, wine and a variety of food products. We recently isolated from OTA contaminated soil vineyard a novel free-living strain of *Acinetobacter* sp. *neg1*, ITEM 17016, able to degrade OTA into the non-toxic catabolic product ochratoxin α. Biochemical studies suggested that the degradation reaction proceeds via peptide bond hydrolysis with phenylalanine (Phe) release. In order to identify genes responsible for OTA degradation we performed a differential gene expression analysis of ITEM 17016 grown in the presence or absence of the toxin. Among the differentially expressed genes, six peptidases up-regulated at 6 h were identified. The degrading activity of the carboxypeptidase PJ_1540 was confirmed *in vitro* in a heterologous system. The enrichment analysis for Gene Ontology terms confirmed that OTA degradation proceeds through peptidase activities and revealed the over-representation of pathways related to Phe catabolism. These results indicate that Phe may represent an energy source for this *Acinetobacter* sp. *neg1* strain and that OTA degrading reaction triggers the modulation of further catabolic activities.

## Introduction

Ochratoxin A (OTA) is a mycotoxin produced by several species of *Aspergillus* and *Penicillium*, which contaminates the food and feed chain world-wide. *Aspergillus carbonarius*, *A. ochraceus*, *A. alliaceus*, *A. steynii*, *A. westerdijkiae*, *A. niger*, *Penicillium nordicum* and *P.* *verrucosum* are considered the main OTA producers (Pitt, 1987; Abarca et al., 1994; Bayman et al., 2002; Frisvad et al., 2006). Cereals and cereal products (Araguás et al., 2005; Makun et al., 2007; Duarte et al., 2010), coffee, grapes, wine (Battilani et al., 2004), dried vine fruits (Covarelli et al., 2012), coffee beans (Taniwaki et al., 2003), spices (Romani et al., 2000), but also livestock products, such as milk (Breitholtz-Emanuelsson et al., 1993) and meat (Perši et al., 2014) are the main sources of human OTA exposure (Miraglia and Brera, 2002).

With regards to the chemical structure, OTA is a phenylalanine-dihydroisocoumarine derivative, composed of a 7-carboxy-5-chloro-8-hydroxy-3,4-dihydro-3-R-methylisocoumarin (ochratoxin α - OTα moiety) moiety and a L-β-phenylalanine molecule (Phe), which are linked through the 7-carboxy group by an amide bond.

OTA is primarily known for its nephrotoxic effects. It has been extensively demonstrated that it is the most causal determinant of porcine nephropathy, as well as of human endemic Balkan nephropathy and chronic interstitial nephropathy in North Africa (Pfohl-Leszkowicz and Manderville, 2007; Stefanović and Polenaković, 2009). It is classified by the International Agency for Research on Cancer (IARC) as possibly carcinogenic to humans (group 2B; IARC, 1993). Furthermore, in 1998 the Scientific Committee on Food (SCF) concluded that OTA has carcinogenic, nephrotoxic, teratogenic, immunotoxic and possibly neurotoxic properties (SCF, 1998).

Due to the toxin stability to both temperature and hydrolysis, the current strategies to control OTA levels are mainly preventive, based on pre-harvest management, and aimed to avoid fungal colonization.

Post-harvest strategies are classified into physical, chemical or biological (Varga et al., 2010). Although the first two are generally reported as effective in reducing OTA levels, they induced relevant changes in sensory and nutritional qualities of food. Moreover, toxic residues in the final products may pose safety concern.

For these reasons, biological methods have been increasingly considered as an alternative to physical and chemical treatments. Numerous bacteria, protozoa, and fungi are able to degrade OTA. Also purified enzymes, including lipases from *Aspergillus niger*, carboxypeptidase A and some commercial proteases have been reported to perform this reaction (Abrunhosa et al., 2010). OTA degradation may occur via two kinds of pathways: the hydrolysis of or the lactone ring or the amine bond, by peptidase activity, that links the Phe to the OTα moiety.

In the first case, the final degradation product is an opened lactone form of OTA, which is of similar toxicity to OTA. The second mechanism can be considered a real detoxification pathway because leads to two non-toxic catabolic products: Phe and OTα. The essential non-toxicity of OTα was demonstrated both in prokaryotic and eukaryotic cellular systems as well as in animal models (Xiao et al., 1996).

We recently isolated from vineyard soils a novel free-living strain of *Acinetobacter* sp. *neg1*, ITEM 17016, capable of degrading OTA into the non-toxic catabolic product OTα. ITEM 17016 genome was sequenced and the phylogenomic analysis placed *A*. sp. *neg1* as sister to *Acinetobacter gyllenbergii* (Fanelli et al., 2015).

Numerous peptidase-encoding genes are present in the genome of ITEM 17016. In order to develop biotechnological applications of the enzymatic degrading activities we described the identification of potential candidate genes and pathways involved in OTA degradation through comparative transcriptional analysis. Moreover, we cloned the identified selected peptidase in a heterologous system and tested the detoxification capability of the recombinant protein.

## Materials and Methods

### Strain

The *Acinetobacter* sp. *neg1* strain used in this study was ITEM 17016 from Agri-Food Toxigenic Fungi Culture Collection of the Institute of Sciences of Food Production, CNR, Bari^1^. It was isolated from a soil sample in a Negroamaro vineyard in Cellino S. Marco (BR, Italy), reported as contaminated by high levels of OTA in previous years (Somma et al., 2012). ITEM 17016 was demonstrated to be capable of degrading OTA *in vitro* (De Bellis et al., 2015; Fanelli et al., 2015).

### Growth Curve and OTA Degradation Assay

Bacteria were precultured overnight. A bacterial suspension was inoculated in minimal medium peptone (MMP) medium [2.5 g/l K_2_HPO_4_ (Baker, Deventer, Holland), 1.0 g/l (NH_4_)_2_HPO_4_ (SIGMA, Steinheim, Germany), 0.2 g/l MgSO_4_^∗^7H_2_O (Carlo Erba, Rodano (MI), Italy), 0.5% Bacto Peptone (Biolife, Milano, Italy)] and cultured at 28°C with shaking at 120 rpm up to Optical Density (OD) of 0.5. A spectrophotometer Ultrospec 3100 pro (Amersham pharmacia biotech) was used for the OD_600_ measurement. For the degradation assay, bacteria were inoculated at 2% in a final volume of 5 ml of MMP medium supplemented with OTA (1 μg/ml) and grown for 144 h. Bacterial growth and cell viability were monitored by measuring the OD_600_ and plate counting for each time point (0, 3, 6, 12, 24, 48, 72, 96, 144 h). For the plate counting, serial dilutions of the bacterial suspensions were prepared in 0.85% NaCl (AppliChem, Darmstatd, Germany), 0.1% Tween (Fisher Scientific, Bridgewater, NJ, USA) H_2_O sterile solution and spread-plated on pre-poured PCA plates. Plates were incubated at 28°C overnight and CFU were counted. Each experiment was performed in triplicate. One milliliter of the culture was centrifuged and the supernatant was used for the chemical analysis.

### RNA Extraction and Library Construction

At 6 and 12 h, 1 mL from each triplicate of cultures grown in the presence or absence of OTA was collected by centrifugation at 13,000 rpm for 10 min. Pellets were then resuspended in RNAlater RNA Stabilization Reagent (Qiagen, Hilden, Germany), centrifuged at 13,000 rpm for 10 min and stored at -80° until extraction.

Total RNA was extracted using the RNeasy Mini Kit (Qiagen, Hilden, Germany), according to the manufacturer’s instructions. The quality and the quantity of the RNA sample were analyzed and verified using a NanoDrop1000 spectrophotometer (Thermo Fisher Scientific, Rodano (MI), Italy) and 2100 Bioanalyzer (Agilent Technologies, Santa Clara, CA, USA), obtaining RIN (RNA Integrity Number) values ranging from 7 to 9.

Total RNA from each preparation (about 1 μg) was depleted of ribosomal RNA using the Ribo-Zero Magnetic kit for Gram negative bacteria (Part #15065382, November 2014, Illumina, San Diego, CA, USA) and purified with RNeasyMinElute Cleanup kit (QIAGEN, Hilden, Germania) according to the supplied instructions. The cDNA sequencing libraries for whole transcriptome analysis were prepared using SureSelect Strand-Specific RNA Library Prep for Illumina Multiplexed Sequencing (Version C.0, December 2014, Agilent Technologies, Santa Clara, CA, USA). Briefly, RNA was chemically fragmented prior to reverse transcription reaction. The first-strand cDNAs were synthesized with random hexamer primers and followed by second-strand cDNA synthesis and ends repair. Then, the cDNAs were 3′-adenylated, ligated to the adaptors, amplified, and indexed. The libraries quality was checked by using the Agilent DNA 1000 assay 2100 (Agilent Technologies, Santa Clara, CA, USA) and quantified by using the Nano-Drop 3300 Fluorospectrometer (Thermo-Scientific, Waltham, MA, USA) with the Quant-IT PicoGreen assay kit (Life-Technologies, Carlsbad, CA, USA). All the indexed library produced were subjected to paired-end 2x120 bp sequencing on the Illumina MiSeq platform, generating for each sample approximately 3 M of 120 bp paired-end reads.

### Data Analysis

The quality of raw data was initially checked by FastQC software^2^. Then RNAseq reads were trimmed using Trim Galore^3^ in order to remove low quality regions and adaptors. The cleaned reads were mapped against the reference genome JSZD01 of *Acinetobacter* sp. *neg1* (Fanelli et al., 2015) using GSNAP (Wu and Nacu, 2010). Finally, CuffDiff software, implemented in the CyVerse (iPlant) Discovery Environment, was used to calculate DE genes between the two conditions (Trapnell et al., 2010). Gene Ontology enrichment analysis was performed using the Java-based tool Bingo (Maere et al., 2005), included as a plugin in Cytoscape (Shannon et al., 2003). The hypergeometric test and a FDR-adjusted p-value threshold of 0.05 were used to select significantly enriched pathways.

### Cloning and Expression of Peptidase-Coding Genes

Preparation of genomic DNA from ITEM 17016 was performed according to the method reported by Cappello et al. (2008). Polymerase chain reaction (PCR) was performed with primers designed to amplify the DNA segments encoding the up-regulated genes in the presence of OTA, flanked by *Nco*I and *Xho*I restriction sites, using the *Phusion*^®^ *High-Fidelity DNA Polymerase* (New England Biolabs, Ipswich, MA, USA; **Table 1**). PCR was performed according to the following steps: denaturation for 30 s at 98°C; 35 cycles: 10 s at 98°C, 30 s at 56°C, 30 s at 72°C; final extension for 10 min at 72°C. The obtained amplicon was purified by using the PureLink PCR Purification Kit (Thermo Fisher Scientific, Waltham, MA, USA) and ligated into the cloning vector pCR-II-Blunt-TOPO (Thermo Fisher Scientific, Waltham, MA, USA). *E. coli* competent cells One Shot TOP10 (Thermo Fisher Scientific, Waltham, MA, USA) were transformed with the construct, following supplier’s instructions. Plasmid DNA was extracted using the PureLink Quick Plasmid Miniprep Kit (Thermo Fisher Scientific, Waltham, MA, USA) and the recombinant clones were subjected to sequence analysis using the ABI PRISM 3130 Genetic Analyzer (Applied Biosystems, Foster City, CA, USA). Plasmid DNA was digested with *Nco*I and *Xho*I (Thermo Scientific, USA) and the insert was ligated into the expression vector pET-28a(+; Novagen, Madison, WI, USA) using T4 DNA Ligase (Thermo Fisher Scientific, Waltham, MA, USA) following supplier’s instructions. The construct was used to transform *E. coli* competent cells BL21(DE3) and BL21-CodonPlus(DE3)-RIL (Agilent Technologies, Santa Clara, CA, USA).

**Table 1:** Nucleotide sequences of primers used in this study with *Nco*I (forward primers) and *Xho*I (reverse primers) restriction sites in italics.

Primer	Sequence	Tm
PJ15_1540_forward	5′-AAA*CC****ATGG*TTTATCCTAAAATGCTAGGC**-3′	56°C
PJ15_1540_reverse	5′-AAA*CTCGAG***GAACAAGTTGCTAAAGAACATTTTG**-3′	


Cells carrying the recombinant plasmid were inoculated in 3 mL of Luria-Bertani (LB; Sigma-Aldrich S.R.L., Milan, Italy) broth supplemented with 50 μg/mL kanamycin (Sigma-Aldrich S.R.L., Milan, Italy), 34 μg/mL chloramphenicol (Sigma-Aldrich S.R.L., Milan, Italy) and cultured overnight at 37°C with rotary shaking (250 rpm). One ml aliquot of cell culture was transferred into 9 mL of fresh LB supplemented with antibiotics and cultured 2 h at 37°C with rotary shaking (250 rpm). Isopropyl-β-D-1-tiogalattopiranoside (IPTG; Sigma-Aldrich S.R.L., Milan, Italy) was added to induce the expression of the recombinant protein. Different IPTG concentrations (0.1, 0.5, 1.0, and 2.0 mM), incubation time (2, 4, and 6 h) and temperature (37 and 30°C) were tested. After the induction, cells were harvested by centrifugation at 4,000*g* for 5 min at 4°C. Bacterial pellets and supernatants were kept at -80°C until further analysis. Total proteins from supernatant liquid were precipitated with trichloroacetic acid/acetone (Sambrook et al., 1989) and then analyzed by SDS-PAGE as previously described (Gallo et al., 2012).

### OTA Degradation by Recombinant Peptidases

The degrading activity of the recombinant peptidases was tested as follows: after IPTG induction, bacterial pellets were suspended in prechilled TS Buffer (Tris 0.25 M, NaCl 1.37 M, KCl 0.027 M, Sigma-Aldrich S.R.L., Milan, Italy) and lysed in a French press (thrice at 1700 psi). Cell lysate and supernatant liquid were incubated overnight with 1 μg/mL of OTA at 28°C and the percentage of OTA degradation and OTα production were measured by high-performance liquid chromatography with fluorescence detection (HPLC-FLD) analysis (see section HPLC-FLD Analysis). Each experiment was performed in triplicate.

### Chemical Analyses

#### Chemicals and Reagents

Acetonitrile (ACN), methanol (MetOH) and acetic acid were purchased from Mallinckrodt Baker (Milan, Italy). Ultrapure water was produced by a Millipore Milli-Q system (Millipore, Bedford, MA, USA). Ammonium acetate (for mass spectrometry) was from Sigma-Aldrich (Milan, Italy). Micro Spin Filter Tubes (0.20 μm, regenerated cellulose) were purchased from Phenomenex (Phenomenex, Torrance, CA, USA).

#### Preparation of Standards

Ochratoxin A stock solution was prepared by dissolving the solid commercial toxin (Sigma-Aldrich, USA) in MetOH (1 mg/ml). Appropriate aliquots of the stock solution were brought to dryness and reconstituted with ACN-water-acetic acid (99:99:2 v/v/v) to obtain standard solutions of OTA within the range 0.05–0.10 μg/ml. The standard of OTα was purchased from Biopure (Romer Labs Diagnostic GmbH, Austria) at a concentration of 10 μg/ml in ACN. Aliquots of the stock solution were brought to dryness and reconstituted with ACN-water-acetic acid (99:99:2, v/v/v) to obtain standard solutions of OTα from 0.01 to 0.10 μg/ml.

#### HPLC-FLD Analysis

Decimal dilutions of the supernatants [both from ITEM 17016 and from transformed BL21-CodonPlus(DE3)-RIL cells] were prepared with ACN-water-glacial acetic acid (99:99:2, v/v/v). Samples were filtered using RC 0.2 μm micro spin filter tubes. Fifty microliters were injected into the HPLC apparatus (Technology series 1260, Agilent, Germany) with a full loop injection system. Direct injection of liquid culture was possible since no interfering peaks eluted at retention times of OTα and OTA. The fluorimetric detector was set at wavelengths of 340 nm (excitation) and 460 nm (emission). The analytical column was a Symmetry C18 (5 μm, 150 mm × 4.6 mm; Waters, USA) with a guard column inlet filter (0.5 μm × 3 mm diameter; Rheodyne Inc., USA). The initial composition of the mobile phase was 55% solvent A (water–acetic acid, 99:1, v/v) and 45% solvent B (ACN-acetic acid, 99:1, v/v) and was kept constant for 14 min, then solvent B was linearly increased to 80% in 2 min and kept constant for 4 min. The mobile phase flow rate was 1 ml/min. OTα and OTA were measured by comparing peak areas with calibration curves; the retention times were 9.69 and 11.25 min, respectively. The detection limit was 0.1 ng/ml for OTA and OTα (based on a signal-noise ratio 3:1).

#### HPLC-HRMS Analysis

Structural confirmation of OTα and identification of others possible degradation metabolites were performed by LC-HRMS. Twenty milliliters of supernatants were also analyzed by LC-HRMS according to the procedure described by Gallo et al. (2012). The column was a Kinetex C18 column (100 mm by 2.10 mm, 2.6 μm; Phenomenex, Torrance, CA, USA). The mobile phase was a multistep gradient of water (solvent A) and MetOH (solvent B), both containing 0.5% acetic acid and 1 mM ammonium acetate. The initial composition of the mobile phase was 20% solvent B, after 3 min the proportion was set at 40% and then linearly increased to 63% in 35 min and kept constant for 11 min. HPLC-HRMS analyses were performed on a benchtop single-stage mass spectrometer (Exactive)^TM^ equipped with a heated electrospray ion source (HESI II; Thermo Fisher Scientific, Bremen, Germany), coupled to an HPLC system Accela (Thermo Fisher Scientific, San Jose, CA, USA). The HESI II interface was used in the positive-ion mode, and the scan range was 50.2 to 1,003.0 *m*/*z*, with a resolution power of 100,000 FWHM (full width at half maximum). Other settings were as follows: sheath and auxiliary gas flow rates, 30 and 10 arbitrary units, respectively; sweep gas, 0 arbitrary units; capillary temperature, 300°C; capillary voltage, 4 kV. The Xcalibur software (version 2.1.0, Thermo Fisher Scientific) was used for data acquisition and processing.

## Results

### Growth Curve

**FIGURE 1:**
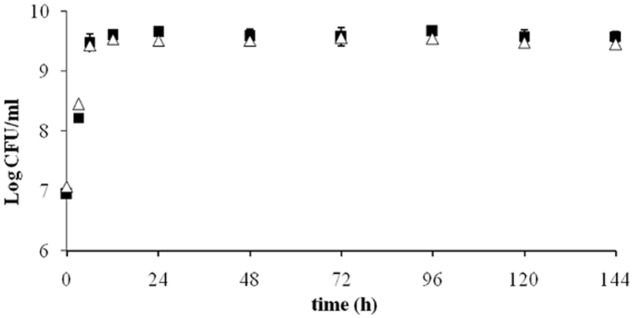
**Viable ITEM 17016 cells in minimal medium peptone (MMP) medium (squares) and MMP supplemented with OTA (1 μg/ml; triangle) at 28°**.

The growth curve of ITEM 17016 in MMP medium and MMP supplemented with OTA is shown in **Figure 1**. The initial inoculum was approximately 7 Log CFU/mL and reached the maximum of 9.6 Log CFU/mL after a 12 h. Number of viable cells slightly decreased after 96 h. Comparison between the two curves did not show notable differences.

### OTA Degradation by ITEM 17016

**FIGURE 2:**
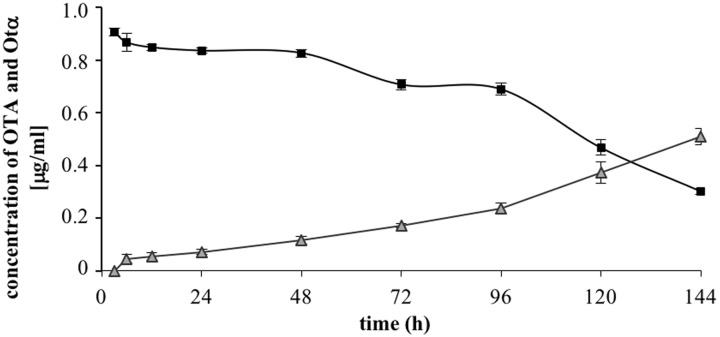
**Changes of OTA (squares) and OTα (triangles) in MMP medium at 28° during the degradation assay**.

The results of OTA degradation by ITEM 17016 are shown in **Figure 2**. OTA level decreased rapidly in the first 6 h of incubation. After this period the degrading activity was slightly reduced until 48 h. The maximum rapidity of OTA decrease, after ITEM 17016 reached its highest viable cell count (**Figure 1**), was measured between 48–72 h and 96–144 h. At 144 h more than 70% of OTA was degraded to OTα. OTα content increased steadily in the medium at the same speed by which OTA was degraded.

### Transcriptional Profiling and Differential Expression Analysis

The analysis pipeline of the RNA seq data has been performed as described in Section “Data Analysis.” A preliminary evaluation of the raw data quality was performed by FastQC software, indicating that more that 95% of reads per sample showed an average quality score higher than 30. Next, TrimGalore software was applied to remove adapters and trim low quality regions at 3′ end of reads using a minimum quality score cut-off of 25. GSNAP software was used to map reads to the reference genome JSZD01 of *A*. sp. *neg1*. The resulting alignment files, in BAM format, were provided to Cuffdiff, which is part of the extensive Cufflinks package developed for the identification of differentially expressed (DE) genes and transcripts (Trapnell et al., 2010). This software estimates the expression changes, controls for variability across replicate libraries and uses the Benjamini–Hochberg procedure to control the False Discovery Rate (FDR). CuffDiff was launched in the Discovery Environment of CyVerse (iPlant Collaborative) interface and platform^4^. The CuffDiff analysis showed 111 and 88 DE genes with a FDR-adjusted *p*-value < 0.05 between OTA-treated and control samples after 6 and 12 h, respectively (**Supplementary Data Sheets 1** and **2**). Among them, six genes code for peptidases and resulted up-regulated at 6 h (**Table 2**). PJ15_1540 and PJ15_2908 both code for a serine-type D-Ala-D-Ala carboxypeptidase similar to the *ASAG1* carboxypeptidase of *Bacillus amyloliquefaciens*, which was reported to degrade OTA *in vitro* (Chang et al., 2015). PJ15_1540 was selected for the experimental validation based on its higher identity (29%) with the *ASAG1* carboxypeptidase.

**Table 2:** Genes coding for peptidases resulted up-regulated at 6 h.

Gene name	Gene ontology molecular function	log2 (fold change)
PJ15_1540	serine-type D-Ala-D-Ala carboxypeptidase activity [GO:0009002]	0.608439
PJ15_2703	metalloendopeptidase activity [GO:0004222]	0.844572
PJ15_2908	serine-type D-Ala-D-Ala carboxypeptidase activity [GO:0009002]	1.02051
PJ15_1202	peptidase activity [GO:0008233]	1.09716
PJ15_2954	serine-type endopeptidase activity [GO:0004252]	0.929888
PJ15_2971	ATP binding [GO:0005524]; ATP-dependent peptidase activity [GO:0004176]; sequence-specific DNA binding [GO:0043565]; serine-type endopeptidase activity [GO:0004252]	0.883469


### Experimental Validation

#### Expression of Peptidase-Coding Genes in *E. coli*

**FIGURE 3:**
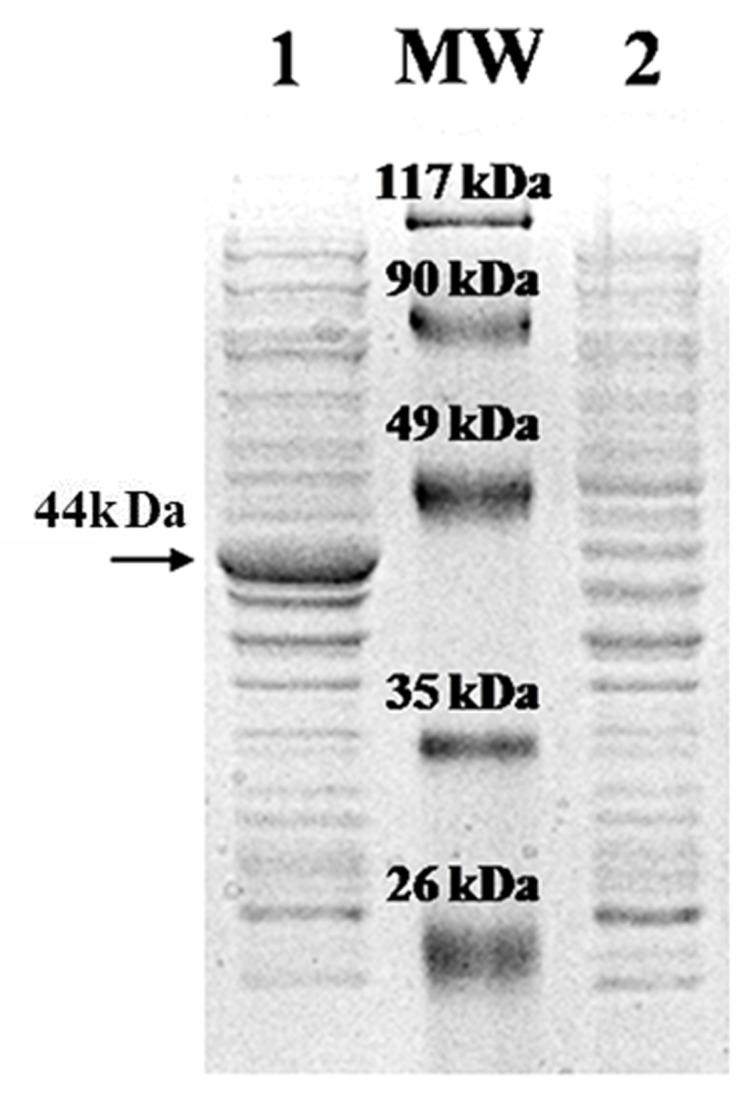
**Expression of recombinant pET-28a(+)-PJ15_1540 in *E. coli* BL21-CodonPlus-RIL.** *Lane 1*: total protein extract of recombinant cell after IPTG induction. *Lane 2*: total protein extract of recombinant cell without IPTG induction. *MW:* Prestained Protein SHARP Mass III (Euroclone). The arrow indicates the recombinant protein.

The amplified DNA fragment of the PJ15_1540 gene showed an electrophoretic mobility on agarose gel corresponding to its theoretical size of 1212 bp (not shown). No mutations were found in the amplicon as compared to the deposited gene sequence. The PJ15_1540 purified fragment was ligated in the vector pET-28a(+) and cloned into *E. coli* BL21(DE3). However, the recombinant plasmid did not accomplish any detectable expression of the protein in this host bacterial strain (not shown). Thus, the construct pET-28a(+)-PJ15_1540 was used to transform *E. coli* BL21-CodonPlus-RIL, which is able to express the tRNA genes for arginine (AGA and AGG), isoleucine (AUA) and leucine (CUA) rare codons. The PJ15_1540 gene was successfully expressed, indicating that the “RIL” host strain was able to promote the recombinant protein production by overcoming the codon usage bias caused by the rare seventh (CUA coding leucine) and fifteenth (AUA) codons present in the gene sequence. To optimize the expression of the PJ15_1540 protein IPTG concentration, time and temperature of incubation were varied. The highest yield of the PJ15_1540 recombinant protein was obtained at 30°C and 2 mM IPTG, after 4 h of incubation. The apparent molecular mass was consistent with the theoretical molecular mass of 44.15 kDa calculated on the basis of the amino acid composition (**Figure 3**).

#### Degrading Activity of the Recombinant PJ15_1540 Protein

**FIGURE 4:**
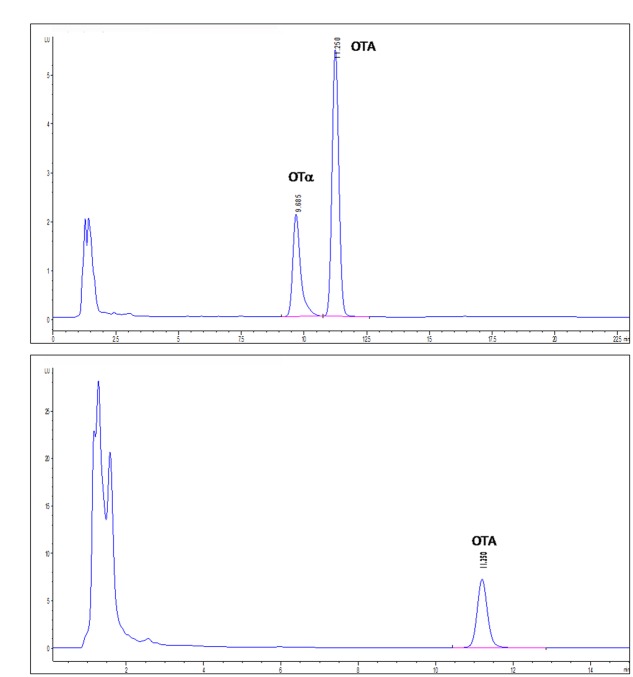
**High-performance liquid chromatography with fluorescence detection (HPLC-FLD) chromatograms of IPTG-induced** **(A)** or not IPTG-induced **(B)** *E. coli* BL21-CodonPlus-RIL-pET-28a(+)-PJ15_1540 cell lysates tested for OTA degradation. Retention times: OTA, 11.250 min; OTα, 9.685 min.

The activity of the recombinant PJ15_1540 protein was tested in cell lysate incubated overnight with 1 μg/mL of OTA. In this condition, PJ15_1540 protein was able to degrade 33% of OTA while a comparable production of OTα was shown (**Figure 4**). The supernatant liquid of induced cells did not show OTA degrading activity. This indicates that the recombinant protein is not secreted into the medium as confirmed by SDS-page (not shown).

### Pathway Analysis

**FIGURE 5:**
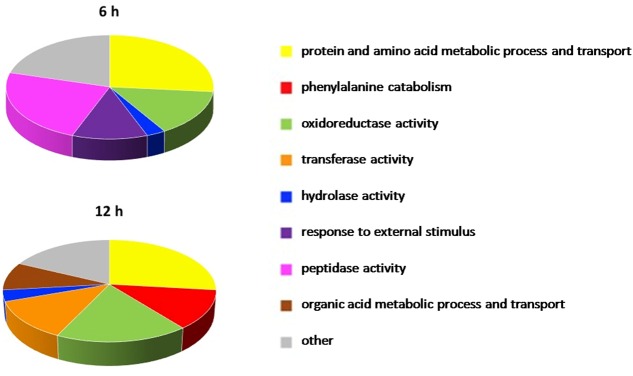
**Pathways significantly dysregulated in the presence of OTA.** Pathways significantly dysregulated at 6 and 12 h are grouped in nine functional categories on the basis of the Gene Ontology relations between terms and manually curated. In the pie charts the arc length of the slices is proportional to the percent of dysregulated pathways of each category.

The enrichment analysis of the DE genes for Gene Ontology terms revealed the over-representation of a total of 59 pathways in 6 h-OTA-grown bacteria and 67 in 12 h-OTA-grown bacteria (**Supplementary Data Sheets 3** and **4**). The dysregulated pathways were grouped in eight functional categories on the basis of the Gene Ontology relations between terms; those pathways not clustering with others were included in a ninth category called *other*. The categories *protein and amino acid metabolic process and transport, oxidoreductase activity, hydrolase activity, response to external stimulus* and *peptidase activity* were dysregulated at 6 h. The categories *protein and amino acid metabolic process and transport, phenylalanine catabolism, oxidoreductase activity, transferase activity, hydrolase activity* and *organic acid metabolic process and transport* were dysregulated at 12 h (**Figure 5**).

## Discussion

This work confirmed the ability of ITEM 17016 to degrade OTA into the non-toxic catabolic product OTα. To investigate enzymes and pathways involved in the OTA degradation process, a comparative transcriptional analysis between bacteria grown in the presence or absence of OTA was performed.

The growth curve of ITEM 17016 was not influenced by the presence of OTA in the culture medium, suggesting that this mycotoxin does not exert any toxic effect on the cells, which is reasonable considering that OTA is present in their natural environment. Almost all the toxin content in the culture medium was converted to the hydrolyzed product OTα. As already demonstrated by previous analysis (De Bellis et al., 2015; Fanelli et al., 2015), the microbial degradation through the hydrolysis of the amide bond results in the Phe molecule and OTα. Our analyses did not reveal the presence of Phe in the medium, thus indicating that this molecule might be immediately used or further degraded by bacterial cells.

Among the up-regulated genes in the presence of OTA, six peptidase-coding genes were considered candidates for the OTA degradation reaction catalysis. PJ15_2703 codes for a metalloendopeptidase which catalyzes the hydrolysis of internal, alpha-peptide bonds in a polypeptide chain. In the reaction mechanism water acts as a nucleophile, one or two metal ions hold the water molecule in place, and charged amino acid side chains are ligands for the metal ions. PJ15_2971 codes for an ATP-dependent serine-type endopeptidase that mediates the selective degradation of mutant and abnormal proteins as well as certain short-lived regulatory proteins. PJ15_2954 codes for the putative serine-type endopeptidase sohB and PJ15_1202 codes for a putative protease. The strongest candidates were PJ15_1540 and PJ15_2908, coding for two serine-type D-Ala-D-Ala carboxypeptidases showing 33% of sequence identity. Indeed, PJ15_1540 and PJ15_2908 proteins showed 29 and 25 identity, respectively, with *ASAG1* carboxypeptidase of *B. amyloliquefaciens*, which was reported to degrade OTA *in vitro* (Chang et al., 2015).

The degrading activity of PJ15_1540 protein was confirmed by heterologous expression in *E. coli*. The recombinant protein was able to degrade OTA into OTα, confirming the same mechanism of hydrolysis of the amide bond demonstrated for ITEM 17016.

In order to investigate at systemic level the OTA effect on the metabolism of the *A*. sp. *neg1* strain, an enrichment analysis of the DE genes for Gene Ontology terms was completed. Consistent with the differentially up-regulated peptidases revealed under OTA presence, genes in the category *peptidase activity* were up-regulated at 6 h of treatment but not at 12 h, indicating an early activation of the mechanism leading to OTA hydrolysis.

After 6 h of incubation with OTA, activities and processes related to the category *response to external stimulus* were dysregulated with respect to the control. This suggested that, in this short period after OTA addition, the strain was sensing the changes in the environment as a stressing condition and activating responses to this stimulus which involves regulation of gene expression and protein activity (Starosta et al., 2014). However, this adaptation was completed within 12 h, as demonstrated by the absence of this category among those which resulted altered after 12 h.

Both at 6 and 12 h molecular functions and biological processes belonging to the *protein and amino acid metabolic process and transport* category were over-represented. In fact, Phe is one of the products of the degrading reaction and this category includes transmembrane transporters and specific amino acid transporters which are downstream the Phe catabolic pathway. Accordingly, among the gene sets displaying significant OTA-related dysregulation at 12 h, seven pathways involved in Phe catabolism were highlighted. This suggests that Phe represents an energy source for ITEM 17016 and that the degrading reaction is followed by the modulation of further catabolic activities. In bacteria phenylalanine catabolism can proceed through different routes (Arias-Barrau et al., 2004; Teufel et al., 2010). Our results confirmed that there are several dysregulated pathways related to these catabolic processes, which are associated with the presence of OTA.

Pathway analysis also shows the alteration of *4-hydroxyphenylpyruvate dioxygenase activity*, which suggests that ITEM 17016 is capable of converting Phe into tyrosine by a pterin-dependent Phe hydroxylase. Then, tyrosine can be converted into 4-hydroxyphenylpyruvate, which is further transformed into 2,5-dihydroxyphenylacetate by a *4-hydroxyphenylpyruvate dioxygenase*. By this way the aromatic ring is then split by a ring-cleaving dioxygenase, with the final production of fumarate and acetoacetate (Arias-Barrau et al., 2004; Teufel et al., 2010).

Phe could be alternatively metabolized via phenylacetate as extensively demonstrated for many bacterial species (Ferrández et al., 1998; Erb et al., 2008; Teufel et al., 2010). The downstream pathways of phenylacetate include the pathway of benzoate degradation (KEGG database^5^). We are led to assume that Phe metabolism in ITEM 17016 might also occur via this route, in accordance with the dysregulation of activities related to benzoate degradation due to the down-regulation of an *hydroxybenzoate 3-monooxygenase*.

The activity *methylmalonate-semialdehyde dehydrogenase (acylating)* related to propanoate catabolism, which is one of the pathways involved in degradation of aromatic compounds (KEGG database^5^), was also altered after 12 h of treatment. In particular, PJ15_0600 gene coding for a *methylmalonate-semialdehyde dehydrogenase* resulted up-regulated with a log2-fold change of 6.8, suggesting a strong involvement of this enzyme in the Phe catabolism. It should be noted that benzoate and propanoate are both organic acids, which is in accordance with the dysregulation of the *organic acid metabolic process and transport* category at 12 h.

Moreover, we reported the over-representation of the *oxidoreductase activity*, which is consistent with the assumption that proteins involved in aerobic catabolism of aromatic compounds are often oxidoreductases (Parales and Resnick, 2006).

## Conclusion

This paper describes a model of workflow for the development of a biotechnological application of enzymatic activities. A traditional microbiological approach combined with a comparative transcriptomic analysis has led to the identification of potential candidate genes and pathways involved in OTA degradation. Finally, the degrading activity of the carboxypeptidase PJ_1540 was validated *in vitro* in a heterologous system demonstrating its potential application in the food chain.

## Accession Numbers

The RNA sequencing raw data were submitted to NCBI under the accession number SRP078981.

## Author Contributions

All authors significantly contributed to this paper. VL, FF, and GM conceived and designed the experiments. VL and FF performed the growth experiment, degradation assays and RNA extraction. MH performed the chemical analysis. CM and CL performed the sequencing. VL and EP performed the differential expression and pathway analysis. GP and MiT supervised the bioinformatics workflow. MaT and FG performed the cloning and expression in heterologous system. All authors reviewed the paper. VL, FF, and AL were responsible for manuscript preparation and GM for the submission.

## Conflict of Interest Statement

The authors declare that the research was conducted in the absence of any commercial or financial relationships that could be construed as a potential conflict of interest.

## Footnotes

1 www.ispa.cnr.it/Collection

2 http://www.bioinformatics.babraham.ac.uk/projects/fastqc/

3 http://www.bioinformatics.babraham.ac.uk/projects/trim_galore/

4 http://www.iplantcollaborative.org/

5 http://www.genome.jp/kegg/

## Supplementary Material

**DATA SHEET 1 | CuffDiff_6h.** Output of *CuffDiff* comparative analysis between OTA-treated and control samples after 6 h of treatment.

**DATA SHEET 2 | CuffDiff_12h.** Output of *CuffDiff* comparative analysis between OTA-treated and control samples after 12 h of treatment.

**DATA SHEET 3 | Pathway analysis_6h.** Output of *Bingo* pathway enrichment analysis of the differentially expressed genes in 6h-OTA-grown bacteria.

**DATA SHEET 4 | Pathway analysis_12h.** Output of *Bingo* pathway enrichment analysis of the differentially expressed genes in 12h-OTA-grown bacteria.
